# Activated Bio-Carbons Prepared from the Residue of Supercritical Extraction of Raw Plants and Their Application for Removal of Nitrogen Dioxide and Hydrogen Sulfide from the Gas Phase

**DOI:** 10.3390/ma14123192

**Published:** 2021-06-09

**Authors:** Aleksandra Bazan-Wozniak, Piotr Nowicki, Robert Wolski, Robert Pietrzak

**Affiliations:** 1 Faculty of Chemistry, Adam Mickiewicz University in Poznań, Uniwersytetu Poznańskiego 8, 61-614 Poznań, Poland; aleksandra.bazan@amu.edu.pl (A.B.-W.); wola@amu.edu.pl (R.W.); pietrob@amu.edu.pl (R.P.); 2 Centre for Advanced Technologies, Adam Mickiewicz University in Poznań, Uniwersytetu Poznańskiego 10, 61-614 Poznań, Poland

**Keywords:** activated bio-carbons, residue of supercritical extraction, pyrolysis, physical activation, toxic gases, adsorption

## Abstract

The waste materials left after supercritical extraction of hop cones and marigold flowers were tested as precursors of activated bio-carbons. Adsorbents were produced by means of the physical (also called thermal) activation method using CO_2_ as the gasifying agent. All the activated bio-carbons were tested for the removal of NO_2_ and H_2_S from the gas phase under dry and wet conditions. The effects of the type of precursor and the activation procedure on the porous structure development, the acid-base properties of the surface, as well as the sorption capacities of the materials produced were also checked. The final products were bio-carbons of medium developed surface area with a basic surface nature, characterized by their high effectiveness in removal of gas pollutants of acidic character, especially nitrogen dioxide (sorption capacities in the range from 12.5 to 102.6 mg/g). It was proved that the toxic gas removal efficiency depends considerably on the sorption conditions and the activation procedure. All materials showed greater effectiveness in gas removal when the process of adsorption was carried out in the presence of steam.

## 1. Introduction

Pollutants of large diversity are emitted to the atmosphere as side effects of human activity [1]. As they are toxic to the environment and humans, the level of their emission and their content in the air have been monitored and in many large cities are known to exceed the safe limits. Most threatened are the developing countries, although in many industrialized countries the level of toxic pollutants in the air is also very high [2]. The toxic effect of air pollutants is felt by man, man’s products, and living natural resources [3,4]. Air pollutants come from anthropogenic and natural sources [5]. The natural ones are volcanic eruptions, fires, lightning discharges, as well as biological processes of cosmic dust [6]. The anthropogenic sources may be divided into four groups: energy production (fuel combustion), transport (land, water, and air), technological processes (chemical industry, metallurgy, oil refineries), and households [7,8,9,10]. The main polluting substances are nitrogen and sulfur compounds, carbon dioxide, and particulate matter [11,12]. Nitrogen compounds make up a numerous group showing a diversity of structures and properties. Among them nitrogen oxides are highly toxic to living organisms, in plants causing leaf withering or sometimes even plant death, while in humans they can be damaging to the lungs and are carcinogenic. The presence of sulfur compounds in the atmosphere hinders the development of living organisms and in humans is very often related to respiratory and cardiovascular system diseases [12,13,14,15,16].

Increasing industrialization leads to increasing concentrations of toxic substances in the atmosphere. One of the many proposed measures to deal with the problem is the use of carbonaceous adsorbents, capable of adsorption of toxic compounds both from the gas [17,18] and liquid [19,20] phase. Carbonaceous adsorbents, especially activated carbons, are commonly used e.g., for air and drinking water purification as well as in wastewater treatment [21,22,23,24]. Precursors of activated carbons may be almost any organic material with a high carbon content [25,26,27]. The growing demand for biocarbon materials along with the depletion of non-renewable resources and the increasing strictness of the environment protection regulations have inspired the search for new precursors of activated carbons. A very promising and increasing group of such new precursors can be assigned to the residues of supercritical extraction of plants or their elements. The literature information on the practical use of waste materials of this type, in particular for production of activated carbons, is scarce [28]. Extraction with a fluid in the supercritical state is effective for obtaining natural dyes, aromas, or taste substances [29,30,31]. For instance, the extract from hop cones is mainly obtained by a supercritical process. It is used not only in the brewery but also in the cosmetic industry as a component of products for skin and hair care. Marigold flowers are an important source of valuable essential oils known to contain lipid compounds of antioxidant properties and desirable for the pharmaceutical and cosmetic industries [32]. According to literature data, extraction with a liquid in a supercritical state of hop cones and marigold flowers is of growing industrial significance so the amount of the waste from the process is expected to increase with time. Unfortunately, at present the waste of this type is usually combusted in power producing plants or furnaces [33]. One of the possible ways of utilization as an alternative to combustion, is their conversion into carbonaceous adsorbents capable of removing pollutants from the liquid or gas phase.

Taking the above into account, the main purpose of this study was to prepare a series of bio-carbons by way of thermal (so called physical) activation of the solid residue generated from the process of supercritical extraction of hops and marigold flowers and to test their ability to remove NO_2_ and H_2_S from the gas stream. The process of adsorption was tested in dry and wet conditions in order to establish the influence of water on the effectiveness of the process. Moreover, the effect of the activation procedure on the physicochemical properties and the sorption capacity of the obtained activated bio-carbons was checked.

## 2. Materials and Methods

The precursors of the activated bio-carbons were solid wastes generated from supercritical extraction of hops (H) and marigold (M) in a powder form with particle size of 0.10–0.80 mm and moisture content of 5.6 wt.% for hops and 4.9 wt.% for marigold. The starting materials were at first pyrolyzed (P) at 600 °C in a nitrogen atmosphere (flow rate of 170 mL/min), for 60 min. The bio-chars prepared (HP and MP) were next subjected to physical activation (Ap) at temperatures of 700 °C (Ap7) and 800 °C (Ap8) in the presence of carbon dioxide (flow rate of 250 mL/min), for 60 min.

For the evaluation of NO_2_ or H_2_S sorption capacity: Bio-carbon samples were placed in a glass tubular reactor of 300 mm in length and 9 mm internal diameter (adsorbent bed volume 3 mL) and tested in dry (D) or wet (W) conditions. A mixture of dry or wet air (of 70% humidity and temperature 22 ± 1 °C) with 0.1% of nitrogen dioxide or hydrogen sulfide was passed through the activated bio-carbon samples at a flow rate of 450 mL/min. The effect of the adsorbent bed pre-humidification on the effectiveness of NO_2_ and H_2_S removal was checked as well. For this purpose, the bio-carbon samples were at first subjected to a wetting procedure for 30 min in an air stream of 70% humidity, a temperature of 22 °C, and at a flow rate of 360 mL/min. Finally, the sorption tests were conducted either in dry or wet conditions, referred to further as mix-dry (MD) or mix-wet (MW), respectively. The NO_2_ or H_2_S concentration was constantly measured with electrochemical sensors—Multi-Gas Monitor Q-RAE PLUS PGM-2000/2020 (RAE Systems, Sunnyvale, CA, USA). Due to the limitations of the operating range of the sensors used, the adsorption tests were stopped at the moment when the breakthrough concentration of toxic gases reached a value of 20 ppm for nitrogen dioxide or 100 ppm for hydrogen sulfide. The sorption capacities of each activated bio-carbon under investigation [mg/g] were calculated on the basis of the nitrogen dioxide or hydrogen sulfide concentration in the inlet gas stream, the breakthrough time as well as the weight of the bio-carbon sample. After the breakthrough tests, the rate of nitrogen dioxide and hydrogen sulfide desorption from the adsorbent bed was monitored. For this purpose the activated bio-carbon samples were exposed to a stream of dry and pure air at a flow rate of 360 mL/min, for a period of 15 min.

The elemental analysis of each bio-carbon sample was performed using a Vario EL III elemental analyzer (Elementar Analysensysteme GmbH, Langenselbold, Germany). The analysis consisted of the catalytic combustion of bio-carbon samples at 1200 °C, the separation of gases on adsorption columns, and their detection based on the difference in thermal conductivity (the repeatability of determinations was over 99.7% for the reference substance). The total ash (mineral matter) content was determined by burning the activated bio-carbon samples in a microwave muffle furnace (Phoenix, CEM Corporation, Matthews, IL, USA) at 815 °C for 60 min. Characterization of the textural parameters of the prepared activated bio-carbons (e.g., surface area, total pore volume, and mean pore diameter) was performed on the grounds of low temperature N_2_ sorption isotherms, measured at −196 °C on a sorptometer Autosorb iQ, provided by Quantachrome Instruments (Boynton Beach, FL, USA). Prior to the isotherm measurements the bio-carbon samples were degassed under vacuum for 8 h, at 300 °C. The surface area of the samples (S_BET_) was evaluated in the range of relative pressure p/p_0_ from 0.05 to 0.30 (according to the Brunauer–Emmett–Teller method). Total pore volume (V) for each bio-carbon sample was calculated on the basis of the amount of liquid nitrogen adsorbed at a relative pressure p/p_0_ = 0.99. The mean pore diameter (D) was calculated from the equation D = 4 V/S_BET_. Moreover, the commonly known t-plot method was applied to determine the micropore volume and area. The pH of each bio-carbon sample was determined according to the following procedure: a portion of 0.2 g of each carbon material was mixed with 10 mL of distilled water and then the suspension was stirred for 12 h, until a state of equilibrium. After that the pH of the suspension was measured using a CP-401 pH-meter (ELMETRON, Zabrze, Poland) equipped with an EPS-1 combination glass body electrode. The content of the surface oxygen functional groups (of acidic and basic character) was determined according to the Boehm method, described in detail in our earlier work [34].

## 3. Results and Discussion

### 3.1. Elemental Composition of the Starting Materials and Carbonaceous Adsorbents Prepared

Table 1 presents the results of elemental analysis of the precursors used and the carbonaceous materials obtained from them, together with the pyrolysis and activation process yield. According to these data, both precursors contained about 50 wt.% of elemental carbon in the structures, indicating their potential suitability for the production of carbonaceous adsorbents [35].

To increase the content of elemental carbon, the solid waste left after supercritical extraction of hop cones and marigold flowers was pyrolyzed at 600 °C. This process brought substantial changes to the structures of both starting materials. The samples HP and MP showed over 20% higher carbon content than the precursors. The increase in C^daf^ content was accompanied by a considerable decrease in the hydrogen and oxygen contribution to the structure, along with small changes in the S^daf^ content. Also the contribution of N^daf^ to the structure increased significantly with respect to that in the precursors. Thermochemical treatment in an atmosphere of neutral gas led also to a considerable increase in the content of ash. As follows from the data from Table 1, the yield of pyrolysis products was at a level of 28–32 wt.% and was a little higher for hops.

Analysis of the collected data also reveals that physical activation leads to further significant changes in the carbon structure. Moreover, both the activation temperature and the type of precursor have an impact on the chemical composition of the materials prepared. All bio-carbons are characterized by a higher content of elemental carbon than the corresponding products of pyrolysis. The highest content of elemental carbon was found in sample HPAp8 obtained by activation of bio-char HP at 800 °C. The content of C^daf^ in the other bio-carbons was at a similar level (71.7–75.1 wt.%). Interestingly, in the bio-carbons obtained from marigold flowers, the content of C^daf^ increased only by about 1% with respect to that in the bio-char MP, which was most probably related to the fact that the carbon matrix of sample MP was much more strongly ordered during the pyrolysis process.

Table 1 data also show that the activation effectiveness depends significantly on the type of precursor and activation temperature. For all bio-carbons the increase in temperature of 100 °C (from 700 to 800 °C) leads to a significant reduction in the yield of the final product; a much greater (by 32 wt.%) decrease is noted for the sorbents obtained from hops. It may be connected to the fact that bio-char HP shows a lower degree of ordering so has a smaller thermal resistance than the sample MP. For the samples obtained from marigold flowers, the decrease in the yield was just 12 wt.%.

Each of the activation products shows a very high mineral admixture content, as the contribution of ash varies from 25.5 to 32.7 wt.%. Such a high contribution of mineral matter in the structure of the bio-carbons prepared suggests that they potentially may be effective for removal of acidic gas pollutants, as indicated in earlier works [36,37].

### 3.2. Textural Properties of the Adsorbents Prepared

Analysis of the data collected in Table 2, reveals that the physical activation of bio-chars HP and MP does not permit effective development of their surface area and pore structure formation. The BET surface area of the bio-carbons varies from only 24 to 183 m^2^/g. Sample HPAp8 is characterized by the strongest developed surface area of S_BET_ = 183 m^2^/g and the total pore volume of 0.15 cm^3^/g, which are both much lower than the values typical of commercial products [38,39]. The reason for such unattractive textural properties of the bio-carbons prepared can be the little difference in the thermal conditions of the pyrolysis and activation processes. A similar dependence has been observed for the carbonaceous adsorbents prepared from olives [40]. Another reason is that a large number of pores can be blocked by mineral substance. Table 2 data also imply that a higher activation temperature (by 100 °C) makes it possible to achieve more favorable textural parameters, especially for the bio-carbons prepared from hops. Sample HPAp8 has over seven times a larger surface area than the analogous bio-carbon activated at 700 °C.

**Figure 1:**
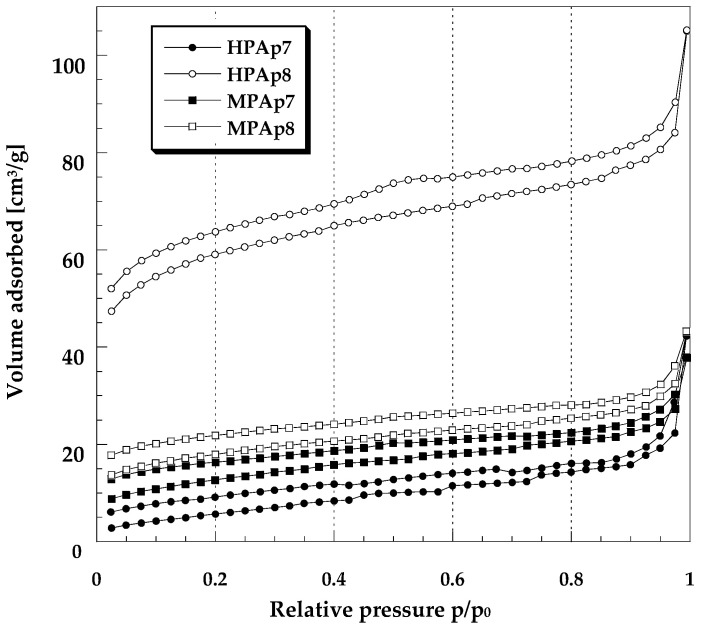
Low-temperature N_2_ sorption isotherms for the bio-carbons prepared from hops and marigold.

The porous structure of the bio-carbons obtained comprises mainly mesopores, as the average pore diameter varies in the range from 3.54 nm to 10.92 nm. The greatest contribution of micropores was found for bio-carbon HPAp8, in which the micropores make up about 67% of all pores. The microporous character of the sample obtained as a result of activation of HP bio-char at 800 °C was also confirmed by the course of the low-temperature nitrogen adsorption/desorption isotherm presented in Figure 1.

### 3.3. Acid-Base Character of the Carbonaceous Adsorbents Prepared

The data characterizing the acid-base nature of the carbon sorbents studied are collected in Table 3. They indicate that the surfaces of the studied materials show basic character as there are no functional groups of acidic nature and their water extract pH values fall in the range 9.6–11.5. The content of basic functional species (e.g., pyrone and chromene structures) depends markedly on the type of starting material and activation temperature. Samples MP, MPAp7, and MPAp8, obtained from marigold flowers, show a much higher contribution of basic species than the analogous sorbents prepared from hops.

The highest number of basic groups was noted for the bio-carbon prepared via thermal activation of sample MP at 800 °C, while the lowest, for bio-char HP. As far as the effect of physical activation temperature is concerned, with its increase the number of basic functional species increases, which is particularly apparent for the bio-carbons obtained from marigold flowers. The basic nature of the surface of the bio-carbons results most likely from the use of CO_2_ as an activating agent, as high activation temperature in combination with carbon dioxide favors the generation of functional groups of basic character [18]. A large number of basic sites on the bio-sorbents’ surface may also be related to a high content of mineral substance in their structures. Analysis of the data from Table 1 and Table 3 shows that with increasing content of mineral matter, the number of basic functional groups present on the surface of the bio-carbons increases.

### 3.4. Adsorption of Nitrogen Dioxide

As indicated by the data presented in Table 4, the efficiency of nitrogen dioxide removal depends first of all on the conditions under which the adsorption test was carried out. The influence of the type of precursor and activation procedure is much less significant. The highest sorption capacities were measured in wet as well as mix-wet conditions. High sorption capacities in these conditions are related to water film formation on the carbonaceous adsorbent’s surface, which favors the capture and bonding of NO_2_ molecules or even nitric acid formation [41]. The impact of the sorption test conditions on the amount of adsorbed NO_2_ is the most pronounced for sample MPAp7, whose sorption capacity after pre-humidification and adsorption in wet conditions is almost 10-times greater than in dry conditions. The highest sorption capacity was established for bio-sorbent MPAp8 whose precursor was marigold flowers. Depending on the variant of adsorption conditions, its sorption capacity changed from 55.3 mg/g (dry conditions) to 102.6 mg/g (mix-wet conditions). According to the data from Table 4, the two samples prepared from hop cones (HPAp7, HPAp8) show similar effectiveness in NO_2_ removal both in the dry as well as mix-dry conditions. For the analogous bio-sorbents obtained from marigold flowers (samples MPApX) much more pronounced differences were observed. For the processes conducted in wet (W) and mix-wet conditions (MW) the differences in NO_2_ removal between the activated bio-carbons obtained at temperature of 700 and 800 °C are significant. As already mentioned, the sorption abilities of the samples towards nitrogen oxide also depend on the type of precursor used for their production. Irrespective of the adsorption conditions, sample MPAp8 is much more effective in removal of NO_2_ than the bio-sorbents produced from the waste left after extraction of hop cones with supercritical CO_2_. No similar relation was observed for sample MPAp7 prepared by bio-char activation at 700 °C. Only in mix-wet conditions did bio-carbon MPAp7 show a higher sorption capacity than samples HPAp7 and HPAp8.

Analysis of the results presented in Table 2 and Table 4 implies that another factor affecting the effectiveness of NO_2_ removal is the degree of surface area development, as indicated by much higher sorption capacities of bio-carbons HPAp8 and MPAp8. This is due to the fact that the larger surface area of activated bio-carbons enables the incorporation of more NO_2_ molecules into the carbon structure [41]. However, the content of mineral substances has a much greater impact on the sorption ability of the carbon materials studied (Table 1). As follows from our earlier studies [37] the presence of mineral substances has a beneficial impact on the effectiveness of removal of acidic toxic gases. Most likely, we are dealing with a reaction between the mineral substance (which consists of alkaline compounds) and gas molecules of an acidic nature. What should be noted is that the bio-sorbents obtained by thermal activation of the post-extraction residue, despite having rather unattractive textural parameters, are more effective in NO_2_ removal than the carbon sorbents produced via chemical activation of plum stones [42]. This information is very important from the economical point of view as physical activation is much cheaper than chemical in the production of carbon adsorbents, especially on the industrial scale. Interestingly, the carbons presented in [42] were characterized with a much better developed surface area and porous structure than the bio-carbons obtained from hop cones and marigold flowers. A probable reason for the much lower sorption capacities of the activated carbons obtained from plum stones is the remarkably microporous character of their structure and the presence of a large number of acidic functional groups on their surface, which are not conducive to NO_2_ sorption.

**Figure 2:**
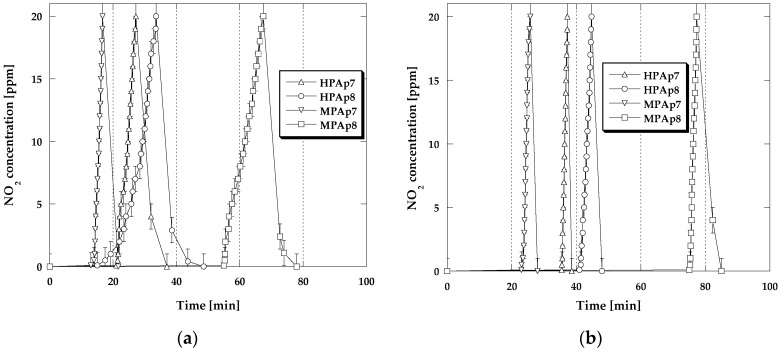
Changes in the NO_2_ concentration during sorption: (**a**) in dry conditions; (**b**) in mix-dry conditions.

**Figure 3:**
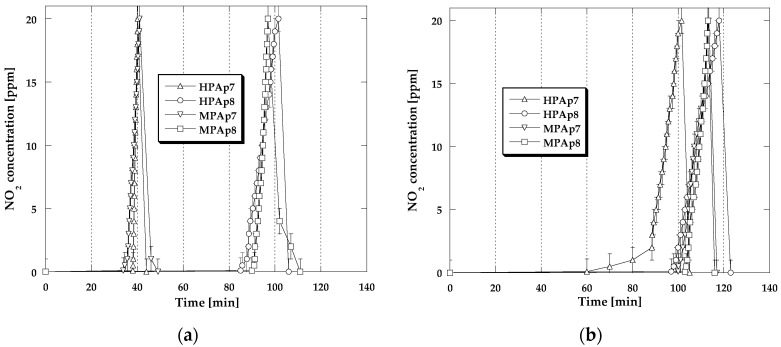
Changes in the NO_2_ concentration during sorption: (**a**) in wet conditions; (**b**) in mix-wet conditions.

For a more detailed analysis of the processes occurring in the bio-carbon bed during sorption tests conducted with the four variants of conditions, Figure 2 and Figure 3 present the NO_2_ breakthrough curves, which illustrate time changes in the NO_2_ concentration (during the adsorption and desorption step). The differences in the curves recorded for the analyzed samples are very small, which means that the mechanisms of NO_2_ sorption are similar.

For all adsorbents studied, for a certain period of time, the nitrogen dioxide concentration in the exhaust gas stream was zero, then after the so-called breakthrough of the bed, it started to gradually increase. Comparison of the curves presented in Figure 2b and Figure 3a,b reveals that preliminary wetting of the bio-carbon bed prior to the test as well as the sorption of nitrogen dioxide in the presence of steam cause the increase in the time period for which the concentration of NO_2_ is equal to zero, thus confirming the beneficial effect of water on the obtained sorption capacities. After cutting off the influx of toxic gas to the adsorber, for all bio-sorbents a rapid decrease in the NO_2_ concentration takes place for 10–15 min (desorption curves are steep), which means that the significant part of the adsorbed NO_2_ has been strongly bonded to the adsorbent structure [18].

**Figure 4:**
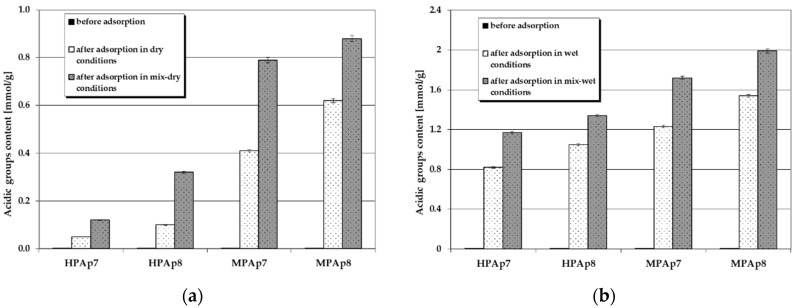
Changes in the content of acidic functional species as a result of NO_2_ sorption: (**a**) in dry/mix-dry conditions (**b**) in wet/mix-wet conditions.

**Figure 5:**
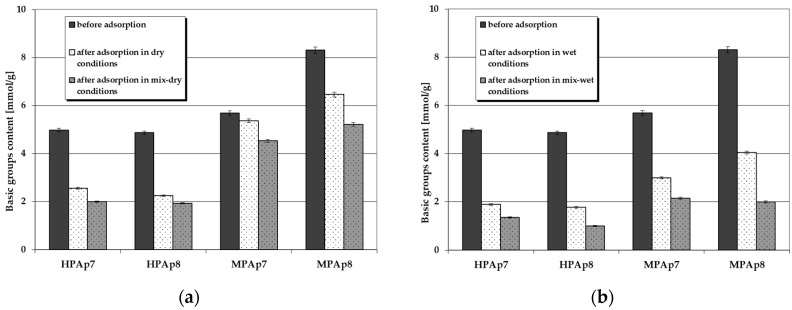
Changes in the content of basic functional species as a result of NO_2_ sorption: (**a**) in dry/mix- dry conditions (**b**) in wet/mix-wet conditions.

Figure 4 and Figure 5 illustrate the changes in the content of acidic and basic functional groups occurring as a consequence of NO_2_ sorption on the bio-carbons. According to these data, on the surface of the carbon materials obtained from the post-extraction residue containing no functional groups of acidic character, after NO_2_ sorption, such species appeared in considerable numbers (especially for samples prepared from waste left after supercritical extraction of marigold flowers). Moreover, their content on the sorbent surfaces was much greater when the test was conducted in wet conditions, in particular after pre-humidification of the bio-carbon bed. As far as the functional species of basic character are concerned, for all activated bio-carbons after sorption of NO_2_, their content significantly decreased, Figure 5.

Table 5 presents a comparison of the obtained results with the relevant literature data. Ebrahim and Bandosz [41] tested adsorption of NO_2_ on zirconium–carboxylic ligand-based porous materials modified with –NH_2_ groups. The most effective in removal of this toxic gas was the sample modified with urea, whose sorption capacity reached 154 mg/g. The most effective from our bio-carbons, MPAp8, showed a sorption capacity towards NO_2_ over 50 mg lower. Comparison of the adsorption/desorption curves reported by Ebrahim and Bandosz with those obtained for our samples reveals significant differences, indicating a different mechanism of NO_2_ sorption. For the sample modified with urea, the breakthrough was observed at the very beginning of the adsorption test and then an increase in the NO_2_ concentration took place. Moreover, starting from the concentration of 15 ppm, the increase in the nitrogen dioxide concentration was very slow, which can explain the greater sorption capacity of this sorbent in comparison with that of bio-carbon MPAp8. For our sample MPAp8, the zero concentration of NO_2_ was maintained for about 100 min and then it rapidly increased to a limiting value of 20 ppm. The desorption curves for the samples studied by Ebrahim and Bandosz are very steep, which points to a strong bonding of NO_2_ on the surface. However, it should be noted that much less effective in nitrogen dioxide adsorption than sample MPAp8 were for example activated carbons prepared by ammoxidation (oxidative ammonolysis) and chemical activation of sub-bituminous coal [43] as their sorption capacities ranged between 26.2 and 66.8 mg/g. Another important point is that the syntheses of carbon adsorbents described in [41,43] is a much more time-consuming and expensive process than preparation of the bio-carbons we prepared.

Kazmierczak et al. [44] used low quality hay, a post-agricultural waste material, for production of carbon adsorbents. The precursor was pyrolyzed at 500–700 °C and activated with CO_2_ in a microwave furnace. According to the results of adsorption tests, higher sorption capacities towards NO_2_ were obtained in the presence of steam in the inlet gas stream. However, the maximum sorption capacity was only 33.7 mg/g, which is much lower than that of bio-carbon MPAp8. Moreover significant differences were observed between the mechanism of NO_2_ sorption by the carbonaceous adsorbents prepared via microwave assisted activation and our bio-carbons. The sample obtained from low quality hay showed a fast breakthrough followed by a slow increase in the NO_2_ concentration to the limit value of 20 ppm. For our bio-carbon MPAp8 the curve presenting the changes in nitrogen dioxide concentration in the exhausted gases with time was quite different.

### 3.5. Adsorption of Hydrogen Sulfide

The bio-sorbents prepared from the residue of supercritical extraction of hops and marigold flowers were also examined for hydrogen sulfide removal. As follows from the data collected in Table 6, they show rather little effectiveness in removal of this toxic gas. Their sorption capacities towards hydrogen sulfide are markedly smaller than for NO_2_ (Table 4). Similar to that of NO_2_ adsorption, the effectiveness of H_2_S removal depends significantly on three factors: the conditions of the adsorption test, the kind of starting material used for activated bio-carbons production, as well as the procedure of its activation.

Unfortunately, the bio-sorbents obtained from the waste left after hop cone extraction showed no sorption abilities towards H_2_S when the test was conducted in the absence of steam (dry conditions). For the other samples, the H_2_S breakthrough capacity varies from 2.9 to 29.6 mg/g of adsorbent, depending on the test conditions. The reason for such low sorption capacities can be the poorly developed specific surface area and the low total pore volume available for adsorbate molecules [44].

The data presented in Table 6 prove that irrespective of the precursor, all activated bio-carbons show much higher sorption capacities when steam is present in the gas stream. This beneficial effect is particularly apparent for the bio-carbons obtained from marigold flowers. The best sorption capacity towards H_2_S was shown by bio-carbon MPAp8, which was also the most effective in NO_2_ removal, while the least effective was sample HPAp7. Moreover, samples HPAp8 and MPAp8 were slightly more effective in H_2_S capture than the analogous bio-carbons activated at a temperature 100 °C lower.

Comparison of the data collected in Table 3 and Table 6 reveals that a high number of basic functional groups present on the bio-sorbent’s surface has a beneficial impact on the sorption capacities towards H_2_S [44]. The samples whose precursor was marigold flowers had more basic functional groups and were able to adsorb more H_2_S than those obtained from hops. This observation is consistent with the correlation found in our earlier work [37].

**Figure 6:**
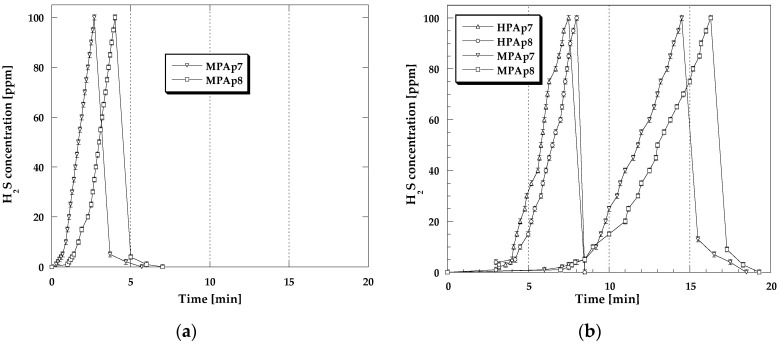
Changes in the H_2_S concentration during sorption: (**a**) in dry conditions; (**b**) in mix-dry conditions.

**Figure 7:**
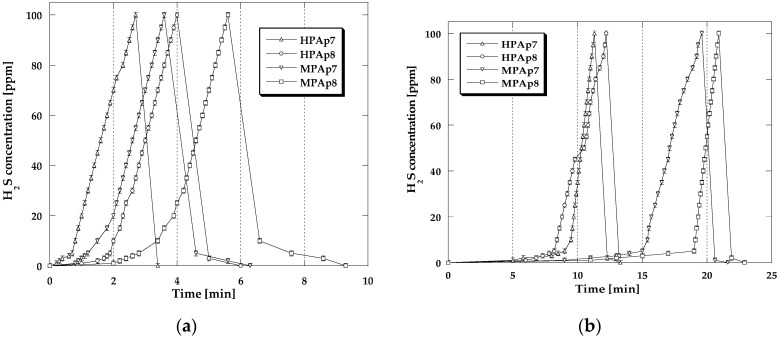
Changes in the H_2_S concentration during sorption: (**a**) in wet conditions; (**b**) in mix-wet conditions.

The shapes of adsorption/desorption curves (Figure 6 and Figure 7) illustrating changes in H_2_S concentration during its sorption have a similar character irrespective of the process conditions. For all the isotherms for a certain period of time, the recorded H_2_S concentration is equal to zero. After the moment of the so-called breakthrough of the adsorbent bed, for all bio-sorbents a rapid increase in H_2_S concentration is observed, especially in dry conditions. Figure 6b and Figure 7a,b show the magnitude of the beneficial impact of the adsorbent bed pre-humidification (water film formation at the bio-sorbent surface) as well as the steam presence in the gas flux on the achieved sorption capacity in relation to H_2_S. The adsorption branches of the isotherms recorded in these conditions have markedly longer sections corresponding to the time when the H_2_S concentration is equal to zero. Cutting off the inflow of H_2_S to the adsorber results in a rapid decrease in its concentration observed within 3–5 min, which means that the majority of this gas has been permanently captured by the activated bio-carbons studied.

**Figure 8:**
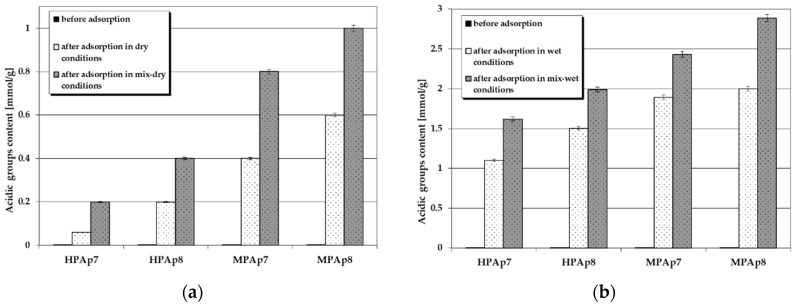
Changes in the content of acidic functional species as a result of H_2_S sorption: (**a**) in dry/mix-dry conditions (**b**) in wet/mix-wet conditions.

**Figure 9:**
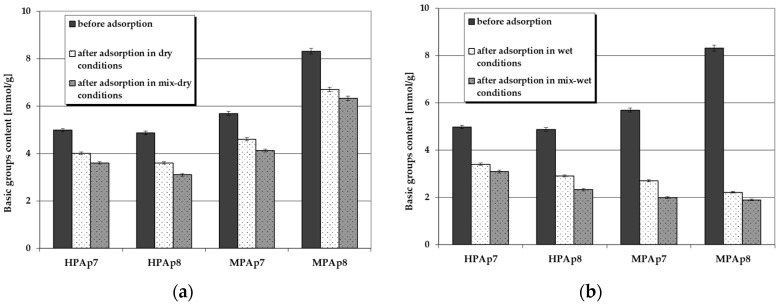
Changes in the content of basic functional species as a result of H_2_S sorption: (**a**) in dry/mix-dry conditions (**b**) in wet/mix-wet conditions.

Figure 8 and Figure 9 present changes in the content of acidic and basic functional groups occurring as a result of hydrogen sulfide sorption on the bio-carbons. After adsorption of this toxic gas the number of acidic functional groups considerably increased. On the other hand, the contribution of the basic functional species decreased significantly (Figure 9), which confirms the interactions between these groups and H_2_S molecules. This decrease is particularly pronounced when the process takes place in wet and mix-wet conditions (Figure 9b).

As indicated by the data given in Table 7 the H_2_S sorption capacities of the bio-carbons studied are much smaller than those obtained for the composite of nano magnesium oxide and granular activated carbon [45]. These nanocomposites are capable of adsorbing from 50 up to 275 mg/g H_2_S, and their sorption capacities depend on the content of magnesium oxide. With increasing contribution of magnesium oxide in the composite, the effectiveness of H_2_S removal increases. It should be also emphasized that the virgin granular carbon is capable of adsorbing 53 mg of H_2_S, which is almost two times higher than the value measured for the most effective of the bio-carbons studied—MPAp8. H_2_S breakthrough capacities of the bio-sorbents studied are also much smaller than those of the activated carbons obtained from low quality hay [44] and coconut shells [46] with the use of microwave heating. In turn, Wallace et al. [47] have used mixtures of sewage sludge and fish waste of various compositions for H_2_S removal. These materials were subjected to pyrolysis and activation by CO_2_. Their sorption capacities varied from 3.2 to 87.1 mg/g H_2_S. The maximum sorption capacity of the bio-carbons studied in this work was nearly three times lower than the highest capacity reported by Wallace. Furthermore, these authors showed that the activation of adsorbents with CO_2_ has a negative effect on H_2_S adsorption.

## 4. Conclusions

The above-described results undoubtedly proved that post-extraction residue can be successfully applied for the preparation of activated bio-carbons. It was shown that physical activation with CO_2_ of precursors of this type leads to bio-carbons of poorly developed porous structure and a basic surface nature. As proved in the adsorption tests, the bio-sorbents obtained, despite the unfavorable textural parameters, are highly effective in removal of gas pollutants of acidic character, especially nitrogen dioxide. The sorption capacities of the bio-carbons depend considerably on the adsorption conditions, the activation temperature, as well as the type of starting material used for their preparation. The most effective adsorbent was sample MPAp8 whose precursor was the residue from the supercritical extraction of marigold flowers. All the bio-carbons obtained were more effective in removal of gas pollutants when the process of adsorption was carried out in the presence of steam. In view of the above, further studies should be concerned with the optimization of the production of bio-carbons from post-supercritical extraction residues for a more effective development of the porous structure, the search for methods for effective regeneration and safe utilization of the used adsorbents, and the testing of the suitability of the use of other waste materials. A successful solution of these problems should permit the large-scale implementation of carbon adsorbents of this type in the near future.

## Figures and Tables

**Table 1:** Elemental composition of the precursors and carbonaceous adsorbents prepared as well as the yield of the pyrolysis and physical activation steps (wt.%).

Sample	Ash	C^daf 1,2^	H^daf^	N^daf^	S^daf^	O^diff 3^	Yield
H	7.6	47.4	9.6	4.8	0.1	38.1	-
M	9.0	50.0	11.3	3.3	0.1	35.3	-
HP	23.0	69.1	3.9	5.1	0.2	21.7	32
MP	28.3	74.1	1.7	4.2	0.3	19.7	28
HPAp7	25.5	71.7	2.5	4.5	0.2	21.1	80
HPAp8	29.9	82.2	3.0	4.6	0.4	9.8	48
MPAp7	27.2	74.9	2.9	4.4	0.5	17.3	75
MPAp8	32.7	75.1	3.4	5.6	0.5	15.4	63

^1^ dry and ash-free state; ^2^ method error ≤0.3%; ^3^ calculated from the difference.

**Table 2:** Textural properties of the adsorbents prepared.

Sample	BET Surface Area [m^2^/g] ^1^	Micropore Area [m^2^/g]	Total Pore Volume [cm^3^/g]	Micropore Volume [cm^3^/g]	Average Pore Diameter [nm]
HPAp7	24	9	0.05	0.01	10.92
HPAp8	183	161	0.15	0.10	3.54
MPAp7	44	29	0.06	0.02	5.34
MPAp8	57	45	0.07	0.03	4.57

^1^ Error range between 2–5%.

**Table 3:** Acidic-basic properties of the carbonaceous adsorbents prepared.

Sample	pH	Total Acidity [mmol/g]	Total Basicity [mmol/g]
HP	10.4 ± 0.1	0.00 ± 0.00	2.66 ± 0.01
MP	11.5 ± 0.2	0.00 ± 0.00	5.15 ± 0.03
HPAp7	9.6 ± 0.15	0.00 ± 0.00	4.98 ± 0.02
HPAp8	10.3 ± 0.1	0.00 ± 0.00	4.87 ± 0.02
MPAp7	10.9 ± 0.2	0.00 ± 0.00	5.69 ± 0.03
MPAp8	11.3 ± 0.15	0.00 ± 0.00	8.32 ± 0.04

**Table 4:** Effect of adsorption conditions on the efficiency of NO_2_ removal [mg/g].

Sample	Conditions			
	Dry	Mix-Dry	Wet	Mix-Wet
HPAp7	30.1 ± 3.9	34.9 ± 4.5	35.1 ± 4.5	55.3 ± 7.1
HPAp8	36.7 ± 4.7	42.1 ± 5.4	70.4 ± 9.1	95.8 ± 12.4
MPAp7	12.5 ± 1.6	20.0 ± 2.6	31.6 ± 4.1	101.1 ± 13.0
MPAp8	50.8 ± 6.6	51.6 ± 6.7	71.6 ± 9.2	102.6 ± 13.2

**Table 5:** NO_2_ sorption capacity for various carbonaceous adsorbents.

Material/Sample	Maximum Adsorption Capacity [mg/g]	References
HPAp8	101.1	(this study)
MPAp8	102.6	(this study)
zirconium—carboxylic ligand	154	[41]
sub-bituminous coal	66.8	[43]
hay	33.7	[44]
pistachio nutshells	77.4	[18]
plum stones	67	[40]

**Table 6:** Effect of adsorption conditions on the efficiency of H_2_S removal [mg/g].

Sample	Conditions			
	Dry	Mix Dry	Wet	Mix Wet
HPAp7	0.0 ± 0.0	3.7 ± 0.5	10.6 ± 1.4	16.0 ± 2.1
HPAp8	0.0 ± 0.0	5.4 ± 0.7	10.9 ± 1.4	17.1 ± 2.2
MPAp7	2.9 ± 0.4	4.5 ± 0.6	20.3 ± 2.6	27.6 ± 3.6
MPAp8	3.8 ± 0.5	7.0 ± 0.9	22.8 ± 2.9	29.6 ± 3.8

**Table 7:** H_2_S sorption capacity for various carbonaceous adsorbents.

Material/Sample	Maximum Adsorption Capacity [mg/g]	References
HPAp8	17.1	(this study)
MPAp8	29.6	(this study)
composite	275.0	[45]
hay	40.5	[44]
coconut shells	109.3	[46]
sewage sludge/fish waste	87.1	[47]
pistachio nutshells	46.4	[18]
sawdust pellets	6.2	[48]

## Data Availability

Data is contained within the article.
